# Asymmetry between the dorsal and ventral digging valves of the female locust: function and mechanics

**DOI:** 10.1186/s12915-024-01930-0

**Published:** 2024-05-31

**Authors:** Shmuel Gershon, Benny Bar-On, Shai Sonnenreich, Amir Ayali, Bat-El Pinchasik

**Affiliations:** 1 School of Mechanical Engineering, Faculty of Engineering, Tel-Aviv University, Tel-Aviv, 6997801 Israel; 2 Department of Mechanical Engineering, Ben-Gurion University of the Negev, Beer-Sheva, 84105 Israel; 3 School of Zoology, Faculty of Life Sciences and Sagol School of Neuroscience, Tel-Aviv University, Tel-Aviv, 6997801 Israel; 4 Center for Physics and Chemistry of Living Systems, Tel Aviv University, Tel Aviv, 69978 Israel

**Keywords:** Biomechanics, Digging, Finite element method, Structure–function relationships, Geometry

## Abstract

**Background:**

The female locust is equipped with unique digging tools, namely two pairs of valves—a dorsal and a ventral—utilized for excavating an underground hole in which she lays her eggs. This apparatus ensures that the eggs are protected from potential predators and provides optimal conditions for successful hatching. The dorsal and the ventral valves are assigned distinct roles in the digging process. Specifically, the ventral valves primarily function as anchors during propagation, while the dorsal valves displace soil and shape the underground tunnel.

**Results:**

In this study, we investigated the noticeable asymmetry and distinct shapes of the valves, using a geometrical model and a finite element method. Our analysis revealed that although the two pairs of valves share morphological similarities, they exhibit different 3D characteristics in terms of absolute size and structure. We introduced a structural characteristic, the skew of the valve cross-section, to quantify the differences between the two pairs of valves. Our findings indicate that these structural variations do not significantly contribute to the valves’ load-bearing capabilities under external forces.

**Conclusions:**

The evolutionary development of the form of the female locust digging valves is more aligned with fitting their respective functions rather than solely responding to biomechanical support needs. By understanding the intricate features of these locust valves, and using our geometrical model, valuable insights can be obtained for creating more efficient and specialized tools for various digging applications.

**Supplementary Information:**

The online version contains supplementary material available at 10.1186/s12915-024-01930-0.

## Background

Various animals, spanning different phyla and body sizes, possess the ability to efficiently burrow and traverse granular matter [1, 2] in order to acquire camouflage [3], forage for food [4], attract mates [5], escape from heat during the day [6], and protect their offspring [7]. To this end, these animals have evolved specific adaptations in their body shape and dedicated digging organs, exemplifying the “form-follows-function” principle [8]. In this context, the female desert locust (*Schistocerca gregaria*, Orthoptera: Acrididae) is especially interesting, as it possesses a specialized digging mechanism comprising two pairs of digging valves which open and close periodically during digging.

**Fig. 1:**
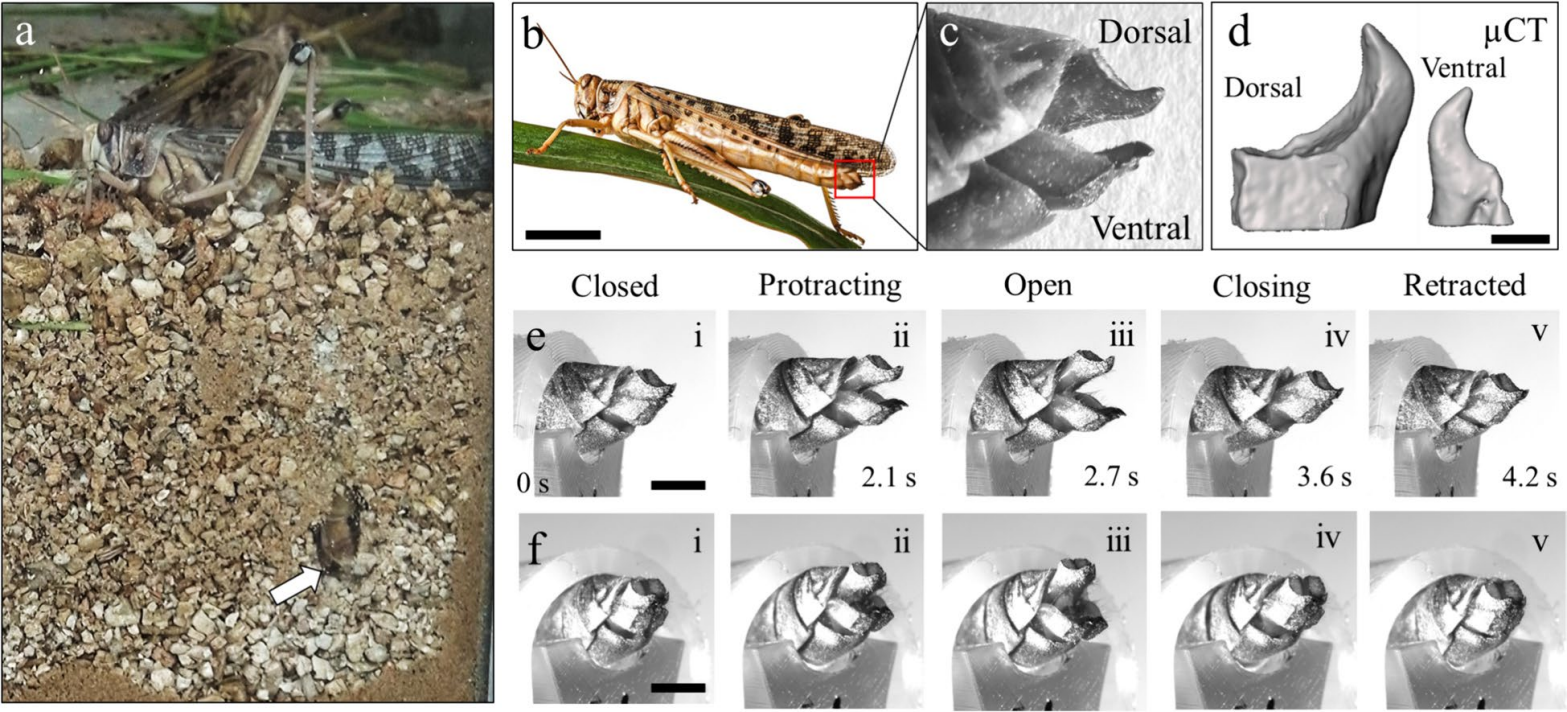
The ovipositor of the female desert locust (*Schistocerca gregaria*). **a** A female locust with its abdomen extended deep into the substrate (a snapshot from a video sequence captured through a glass wall). The red dashed square indicates the end of the abdomen, where the ovipositor digging valves are located. **b** The digging valves are located at the end of the female locust’s abdomen, denoted by a red square. Scale bar corresponds to 2 cm. **c** A higher magnification of the digging valves. **d** A computerized tomography scan of the dorsal and ventral valves. Scale bar corresponds to 1 mm. The digging cycle of the valves from (**e**) side and (**f**) oblique views. Scale bars correspond to 2.5 mm

The ovipositor valves are located at the tip of the female locust abdomen (Fig. 1(a, b); red, dashed square). Figure 1(c) depicts a higher magnification of the two pairs of digging valves, dorsal and ventral. A micro-computerized tomography scan (μCT) of the tips of the dorsal and ventral valves, to scale, is shown in Fig. 1(d). High-resolution μCT scans were used to parameterize and quantify the geometry of the valves. While the dorsal pair and the ventral pair of valves have different shapes, each pair is bilaterally symmetrical between left and right.

During nymphal development, the female locust oviposition valves undergo a process of differentiation and gradual enlargement spanning five nymphal stages [9–11]. By the time of the final molt into adulthood, these valves are nearly fully developed. Subsequently, they become densely sclerotized over a period of 2 to 3 weeks, culminating in sexual maturation. The ovipositor valves serve the crucial function of enabling the female locust to extend its abdomen deep into the ground, up to approximately 10–12 cm, while extending her abdomen [12], facilitating the laying of eggs in a suitable and secure environment [13, 14]. The two pairs of valves, situated at the tip of the abdomen, carry out the burrowing function through a cyclical motion of opening and closing, around a single axis (Fig. 1(e, f)). The ventral valves appear to serve as a pivot point and pull the abdomen into the ground, while the dorsal valves primarily function to clear away soil [2, 15]. While the intra-abdominal pressure maintains the abdomen stable against spontaneous undesirable retraction [15], the valves are the ones responsible for pulling the abdomen and propagation underground.

Locust digging has been studied using simplified 2D models and simulations [16, 17], and the direction-dependent biomechanics of the locust’s major (dorsal) digging valves were recently quantified and analyzed [14]. However, the distinctive geometric shape of each pair of valves and the effect of this shape on the biomechanical properties have remained elusive. Specifically, since the dorsal valves are considered to have the dominant role in loosening and shoveling the soil out of the way of the extending locust abdomen [2, 16], it was particularly intriguing to understand whether and how their shape differs from that of the ventral valves. Note that the lack of symmetry pertains not to morphological asymmetry (the differences within the pairs of dorsal and ventral valves), but rather to the geometric differences between the dorsal and ventral valves.

Biomechanical research of ample arthropods’ cuticular organs has been conducted, including the spider fang [18], spider claws [19], beetle mandibles [20], bee stingers [21], wasp ovipositors [22], and others. These studies have established connections between the geometry and structure of the organs and their respective functions [18, 23–25]. Digging organs are particularly interesting due to their ability to serve multiple functions, including the displacement of soil, efficient removal of materials while shoveling, and withstanding external loads during propagation. Understanding the interplay between form and function holds great potential for engineering and bioinspired robotics [26–32].

In this work, we focused on the digging valves of the female locust as a model system for a successful subterranean digging mechanism, a task crucial to the organism’s fitness. We developed a geometrical model depicting the morphological shape of the digging valves and identified key design parameters that distinguish the dorsal from the ventral valves. Employing Finite Element (FE) simulations, we examined the role of the valves’ geometry in their load-bearing stresses to the different external forces exerted during digging. Finally, we analyzed alternative valve “designs,” by altering the geometry of the model beyond that of its natural form, in order to understand the distinct shape of the valves and its impact on their mechanical stability against external loads.

## Results

**Fig. 2:**
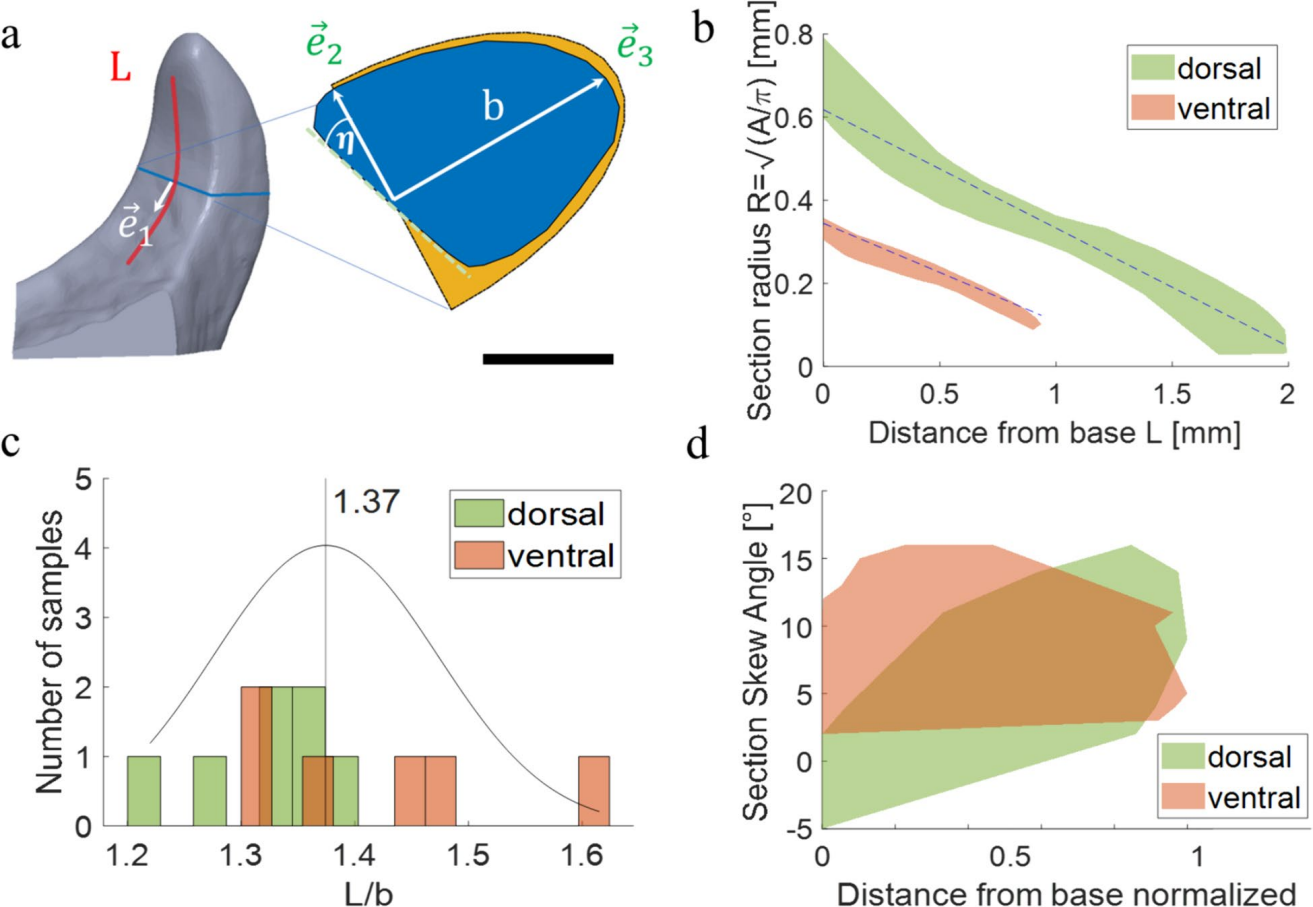
Geometry of the female locust valves. **a** Characteristics of the valves’ cross-section. The centerline of the digging surface is denoted as *L*, and the semi-major axis of the base section of the valves’ tips is denoted as *b*. The local direction of the surface centerline is denoted as ${\overrightarrow{e}}_{1}$. The unit vectors ${\overrightarrow{e}}_{2}$ and ${\overrightarrow{e}}_{3}$ depict the major and minor axes of the semi-elliptical cross-section model. *L* and *b* of the dorsal tip are roughly double the length of *L* and *b* of the ventral tip. A representative cross-section of the valve is shown in blue, while a hemi-ellipse, without a skew, is shown in yellow. The skew defines the slanted shape of the valve’s cross-section in comparison to an ideal hemi-ellipse. Scale bar corresponds to 250 μm. **b** The effective cross-section radius, namely, a radius of an effective circle of the same area, versus the distance from the base of the dorsal (green) and ventral (red) valves. The dotted lines correspond to linear fitting. **c** Distribution of the aspect ratio, *L*/*b*, in dorsal and ventral valves. **d** Distribution of cross-section skew angles

The main geometrical features of the dorsal and ventral valves and their development along the valves are shown in Fig. 2a.

First, we measured the local effective radius, defined as (*cross-section area/π*)^1/2^, along the centerline and fitted a linear function (Fig. 2b). The section size decreases from base to tip at a slope of 0.29 for the dorsal valves and 0.24 for the ventral valves, a statistically insignificant difference. In other words, the cross-section radii of both the dorsal and ventral valves scale almost identically with the distance from the valves’ base, although the cross-section radii of dorsal valves are roughly twice the radii of the ventral valves.

In addition, we measured the ratio between the length of the centerline *L* and the length of the semi-ellipse radius along the major axes. This aspect ratio, *L/b*, reveals whether variations in the slenderness of the valves may indicate differences in their soil penetration abilities. Both valves have an aspect ratio of about 1.37 (*p*-score 0.08; Fig. 2c); hence, they are almost identical, although the dorsal valves’ tips are twice the size of the ventral ones.

We found that in order to adequately characterize the cross-sectional shape, the symmetry of the semi-ellipse must be skewed by an angle, *η*, i.e., the angle by which the minor axis of the semi-ellipse is rotated in relation to the major axis. We assessed the skew angle of all sections along the valves for different samples (*N* = 7 (dorsal) and *N* = 6 (ventral)), calculated the slope for each sample, and performed a *t*-test. We observed a progressive increase in skew along the dorsal valve’s length, while in the ventral valves, the skew remains constant. Figure 2d depicts the distribution of skew angles from the base to the top of the dorsal (green) and ventral (red) valves along their normalized lengths. The skew angle of the dorsal valves increases from a mean value of 3° at the base up to 13° at the tip, while the ventral valves display an approximately constant skew of about 9°.

**Fig. 3:**
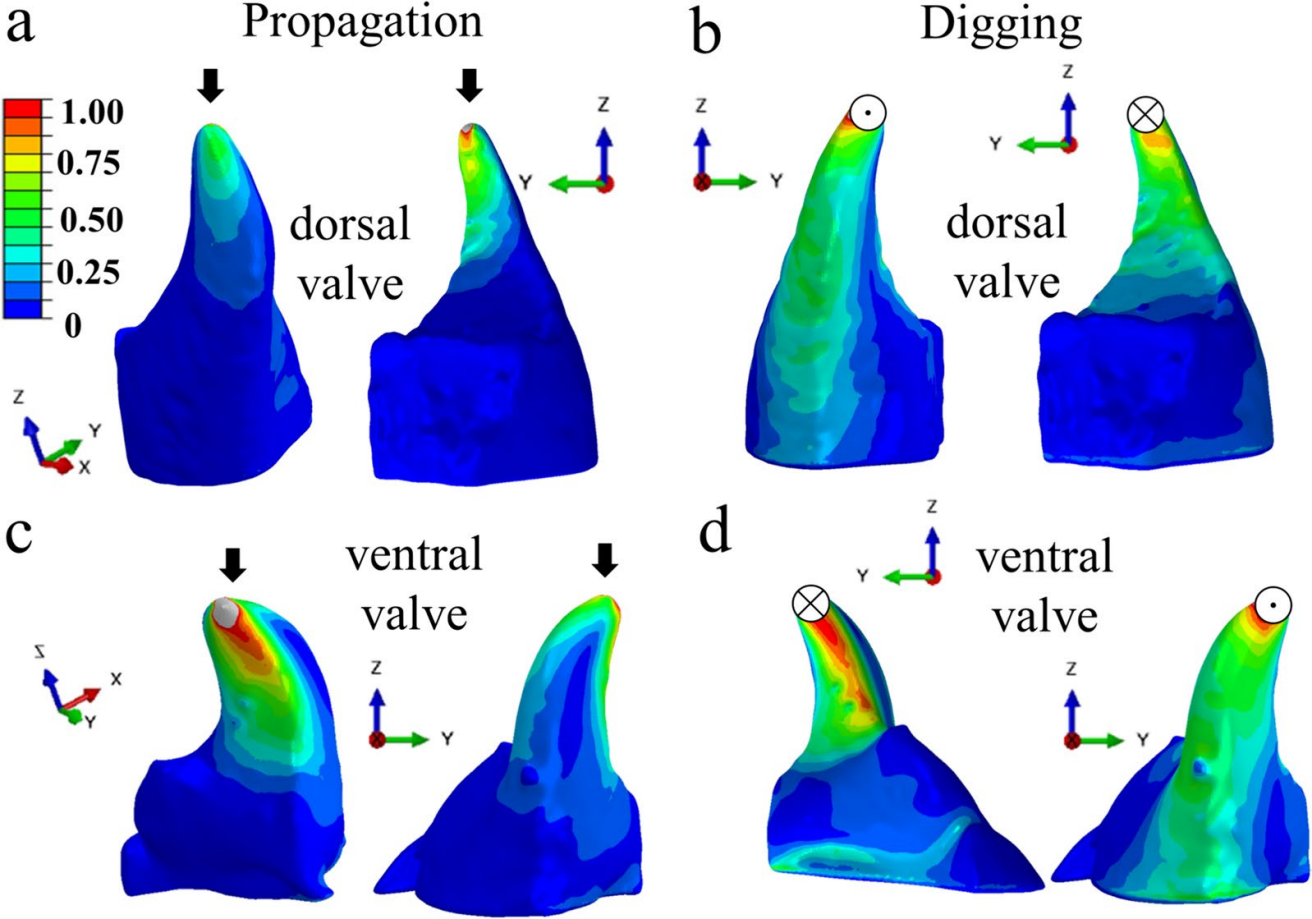
Normalized von Mises stress distribution in the dorsal (top) and ventral (bottom) valves in response to external tip-force loadings. **a**, **c** The propagation direction. **b**, **d** The digging direction. Each configuration is shown from the medial (inner) and the outer perspectives

We then employed the finite element method (FEM) to simulate the mechanical response of the dorsal and ventral valves to applied forces. The stress distributions in the valves in response to external forces in the two main directions of operation, namely propagation and digging underground, are shown in Fig. 3. In both the ventral and the dorsal valves, the load is mostly distributed throughout the outer surface of the valves in the propagation direction. However, in the digging direction, the stress is distributed on both the medial (inner) and the outer surfaces. In both directions, the loads in the dorsal valves are 30–40% lower than in the ventral valves.

**Fig. 4:**
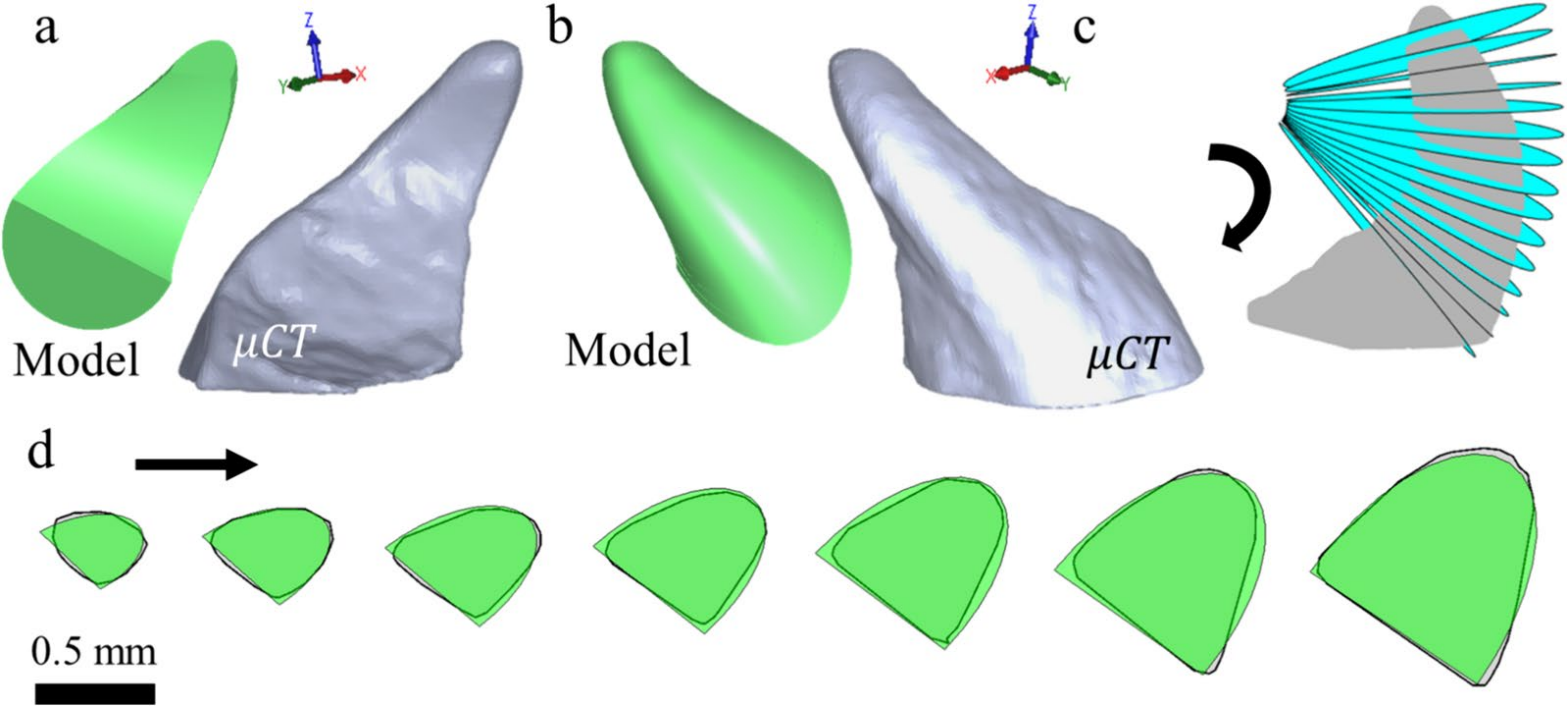
Mathematical model of the locust digging valves. The analytical model (green) and a three-dimensional μCT scan (gray) of a dorsal valve from **a** outer and **b** medial perspectives. **c** Sectioning of the μCT model at equal distances throughout the centerline. **d** Overlay of cross-sections of the valve model (green) and the μCT scans (gray)

Next, we constructed a geometrical model of the female locust valves. Semi-elliptical sections were fixed to a Frenet-frame [33] along a fitted centerline, resulting in a three-dimensional shape [34–39]. The dimensions and skew (defined as the shift from symmetry of the semi-elliptical cross-section, as previously discussed in Fig. 2d) of these semi-elliptical sections were adjusted in a linear manner to match the skew and size obtained from the scanned valve sections. Figure 4a and b depict the resulting three-dimensional model of a dorsal valve (shown from the dorsal and ventral perspectives). Figure 4c depicts an equidistant sectioning of the dorsal valve, and Fig. 4d depicts the natural cross-sections of the dorsal valve overlaying the geometrical model. Equidistant sections are presented in the range of 0 and 0.98 mm from tip to base.

**Fig. 5:**
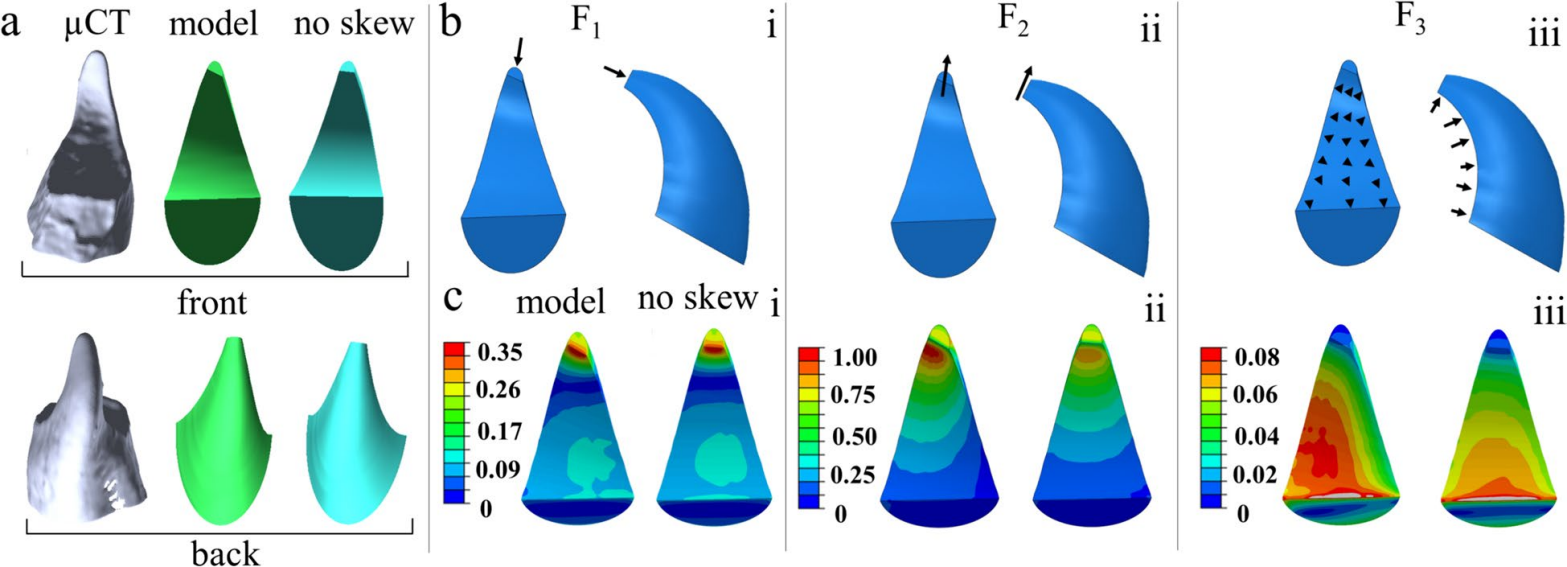
Mechanical response of valve models with varying shapes. **a** Computerized tomography scan of the dorsal valve (left), a model of the valve (middle), and the model without the skew (right). **b** Applied forces in the direction of (i) digging (F_1_), (ii) propagation (F_2_), and (iii) uniform distributed force (black arrows). **c** Simulations showing the von Mises stress distribution in the valve model (left) and the model without the skew (right). For each load case, a normalized stress scale bar is presented

Finally, we investigated the effect of the valves’ distinct geometric features on the stress distribution (Fig. 5) by altering the geometry of the model beyond that of its natural form. Specifically, we compared a model fitted to the natural dorsal valve, to the same model with nullified skew in all sections (Fig. 5a). The skew in the modeled valve was set to linearly increase from zero, at the base of the valve, and up to 32° at the tip.

Figure 5b depicts the force loadings applied on the valve models: namely, tip-forces in the (i) digging (F_1_) and (ii) propagation (F_2_) directions. We also examined a third load of equally distributed force (total force F_3_) on the internal surface of the valves during digging. The overall total forces F_1_, F_2_, and F_3_ are equal. Figure 5c presents the FE results—manifesting the stress distributions in the two models under the different loads.

While we discuss and compare the normalized stress distributions in scans of the real valves (Fig. 3) and in the modeled valves (Fig. 5), we note that the simulations of the modeled valves produced stress patterns and deflections that are comparable to those observed in simulations conducted on the scans of the real valves subjected to F_1_ and F_2_ forces (Fig. 3). Specifically, in the simulations of the modeled valves, the maximal stress was roughly 1.9 MPa for ${\text{F}}_{1}$ and 4.5 MPa for ${\text{F}}_{2}$, while in the scanned biological valves for both load applications, the maximal stress was roughly 3 MPa. Figures 5c (i) and (ii) show that the significance of the skew is not substantial under forces applied at the tip of the valve (F_1_ and F_2_). Yet, the skewing causes the stress concentration near the tip to shift from the center of the valve to the side Fig. 5c (iii). Even though the stress is roughly identical under the forces F_2_ and F_1_, when F_3_ is applied, the stress in the center increases by approximately 20–30%. The difference between the normalized von Mises stresses at the proximal surface, which does not come in contact with the soil during digging, is presented in Additional file 1: Figure S4.

In the analysis of the scanned valves, we have excluded regions of very high-stress concentrations at the tips, where loads are applied, as these are considered mathematical artifacts, yielding the same maximal value. Conversely, in the case of the model valves, these are mathematically pristine surfaces where distributed loads can be applied, eliminating the need for such filtering. These stress values are intended to demonstrate qualitatively that the results from the finite element analysis (FEA) of both the scanned and modeled valves are of similar magnitude. Another factor contributing to the differences is the presence of inner cavities and imperfections in the scanned valves, which are not accounted for in the mathematical model.

## Discussion

The female locust digging valves constitute a unique digging apparatus. The present study used *Schistocerca gregaria* as a typical locust. It is not unique in its morphology—many species of locust dig holes in order to lay their eggs and have similar adaptations of the ovipositor [1, 40]. The dorsal valves propagate and compact soil to create the hole, while the ventral valves function as anchoring tools while the abdomen is pulled into the ground. They mostly share a common shape, albeit with a few key differences.

The cross-section radii of both the dorsal and ventral valves scale linearly with the distance from the valves’ base, although the cross-section radii of dorsal valves are roughly twice the radii of the ventral valves (Fig. 2b), probably to increase the surface area available for shoveling. The two valves possess the same aspect ratio of length over base width (*L/b*, Fig. 2c). In addition, we defined a skew angle to describe the deviation of the valve cross-section from that of a semi-ellipse. While the skew remains roughly constant along the ventral valves, it increases along the dorsal valves. We suggest that this constant skew of the ventral valves creates a concave area that presses better against the soil, while the increasing skew of the dorsal valves contributes to facilitating penetration into the ground and more efficient removal of soil. In other words, the increasing skew along the dorsal valves can be attributed to the need to generate soil flow and compression towards the center of the valve base, while in the case of the ventral valves, no flow needs to be created but, rather, a stable pressure point. Specifically, skewing assists in concentrating the pressure exerted by the ventral valves. Without skewing, the valves would evenly distribute pressure on the soil, resulting in no concentration of stress. Consequently, there would be no creation of an area compressed by pressure from both valve tips.

Das et al. [14] have shown that the dorsal valves possess much more structural resilience to load than that required to withstand the external forces exerted during oviposition digging throughout the locust’s lifetime. Here we asked whether the structural differences between the dorsal and ventral valves influence their mechanical stability against failure due to external loads during digging. Our finite element simulations reveal that the ventral valves are subjected to higher stresses, roughly twofold in some areas, than the dorsal valves, both in the propagation and digging directions. This is explained by the ventral valves’ smaller cross-section. Yet, the maximal von Mises stress is in the same order of magnitude in both the dorsal and ventral valves and corresponds to approximately 3 MPa. Consequently, their structural differences do not support the assumption regarding differences in the valves’ mechanical stability against external loads. Namely, the stress developed in the valves in response to external loads is in the same order of magnitude (Fig. 3). Therefore, the structural differences are likely to serve another function, rather than mechanical stability, probably related to the different functions the valves serve.

To examine this hypothesis, we employed a mathematical model that allowed us to create two prototypes. One, with its cross sections progressively skewing, up to an angle of 32°, and a second model without any skewing along its length. This enabled us to isolate the impact of the skew on mechanical stability against external loads. Our finite element simulations of stress distribution in response to applied loads revealed that the presence of skew in the geometry did not significantly alter load distribution when forces are applied at the valves’ tips (F_1_ and F_2_ loads). However, a substantial effect of skew became evident when simulating the application of uniformly distributed pressure to the soil shoveling surface (F_3_ load). This load mimics the interaction between the valve and soil during shoveling and post-penetration of the soil. Hence, introducing skew in the dorsal valve, both increased the stress concentration in the digging surface and shifted it towards the side of the valve, and towards the tip. This may serve in compressing the soil between the valves’ tips, reducing the likelihood of soil slipping through the gap between the tips, by creating common soil failure boundaries [41, 42]. Specifically, each valve displaces a distinctive volume of soil in its vicinity. When valves are in proximity to each other, their respective volumes can overlap, potentially creating a stagnant region within the overlapping volumes, enhanced by the skewed shape of the valves. This intermediate area between the valves may increase the effective shoveling area and enhance the efficiency of soil displacement (see Additional file 1: Figure S5 in the Supporting Information for the open and closed states of the valves). However, the increased stress concentration can compromise mechanical stability, suggesting that skew has evolved to enhance soil-shoveling performance, albeit at the expense of mechanical strength. Yet, the typical exerted forces on the valves during digging are not sufficient to cause breakage throughout the female locust life, as we showed in our previous study [14].

In the case of the ventral valves, there is no gap between the tips, which are skewed at a constant angle towards one another. These features of the ventral valves stand out, in contrast to those of the dorsal valve. The ventral valves do not need to shovel soil but only to create a pressure point in the soil and, thus, do not require an increase in their effective shoveling surface. Hence, although the overall shape of the dorsal and ventral valves bears a resemblance, sharing a comparable aspect ratio, the ventral valves’ tips are discernibly smaller. In addition, they lack any attributes that might enhance their soil-shoveling ability, primarily featuring characteristics designed for local soil compression point generation.

At present, the design of artificial burrowing tools predominantly depends on human experience and expertise, with a limited incorporation of soil mechanics theory, primarily influenced by Coulomb’s work [43]. Although there have been developments in soil-tool interaction calculations concerning tool geometry, these are limited to date to simple plate shapes [44]. Thus, there is untapped potential in the field of burrowing-tool design by exploring the quantification and mathematical representation of shapes derived from nature. From a natural perspective, the valves belong to a class of structural mechanical elements including claws, teeth, fangs, and jaws, which anchor into target materials or tissues [45–51]. All of these experience either tip forces or distributed loads on their contact surface, which promote stresses within the organs. In a broader perspective, in all these systems there is a dedicated organ that provides necessary anchoring in order to complete the biomechanical function, namely, feeding, frictional support, hanging, and soil displacement.

The findings from this study have practical applications in advancing the development of 3D-printed burrowing tools, extending beyond the natural valve shapes. This has the potential to enhance various tool-burrowing characteristics, facilitating adaptability to different soil types. Such an approach holds the promise of enhancing digging efficiency, particularly in terms of soil removal and reduced energy consumption, while maintaining robust mechanical stability against failure. One potential strategy involves creating different pairs of valves, each pair tailored to a specific function. For example, one pair can be designed to increase soil flow and displacement, improving burrowing capabilities, while the other pair can serve as portable anchors to facilitate underground propagation and locomotion. Conducting digging experiments and simulations using these modified shapes can deepen our understanding of the intricate relationship between a tool’s shape and its burrowing function. The emerging field of bio-inspired burrowing robotics provides an exciting platform for testing and integrating these innovative models into practical applications [52, 53].

## Conclusions

The asymmetry between the dorsal and ventral valves of the female locust supports their assigned different functions during oviposition. The dorsal valves play the role of compacting the soil and creating a stable hole. This role is supported by the increasing skew or twisting of the valves, which may enhance the flow of granular matter on the one hand and the compression of displaced grains outwards on the other hand. The ventral valves, however, have a constant skew and mostly perform as anchors, rather than diggers. As such, they are roughly half the size of the dorsal valves and are subject to higher stresses under external loads. These findings may lead to design guidelines for locust-inspired robotic diggers; increasing the efficiency of digging by using a complex mechanism that is based, however, on a simple motion along a single axis.

To summarize, this study confirms the division of function between the dorsal and ventral valves of the female locust. One set of valves digs, and the other stabilizes the digging valve against the side of the hole. This means that the ovipositor valves constitute a roughly independent digging mechanism, requiring no drive from outside, and so can be steerable and make holes in any direction.

## Methods

### Female desert locust oviposition

Several gravid females were housed overnight in a metal cage with a glass wall, extended above as well as below the Vermiculite substrate. Vermiculite is used since it adheres poorly to the glass wall of the cage and allows better visualization of the oviposition. A Sony HD video camera was positioned in front of the glass wall to allow capturing the oviposition digging sequence.

### Imaging of the valves during the digging cycle

For the images in Fig. 1(e, f), a sexually mature female locust was fixed to a custom-built holder using silicone strips. The holder was bound to an adjustable Manfrotto 454 stage to ensure accuracy in positioning the female locust at the center of the camera frame. The ventral connectives between the head and thoracic ganglia were lesioned to activate the digging sequence. The oviposition valves’ digging sequence was captured from two different angles, using Image Systems—TEMA DIC—Digital Image Correlation and two MER2-160-227U3M cameras with 75-mm focal length lenses. The video was captured with a frame rate of 60 fps. External lighting was used, including 2 LED projectors. We examined 3 different locusts. Each video is approximately a minute and a half long, with over 20 digging cycles.

### μCT analysis

Six adult locust females with no oviposition history were scanned. All scans were performed using an X-ray computerized tomography system (XT H 225 ST, Nikon Metrology NV, Leuven, Belgium), operated using a 225 kV 225 W reflection target, utilizing the following scan parameters: isotropic voxel size of 5 μm, 180 kV, 27 μA, without filter, 3141 projections, 500 ms exposure time, at the Shmunis Family Anthropology Institute, Dan David Center for Human Evolution and Biohistory Research, the Faculty of Medicine, Tel Aviv University.

### Geometrical modeling

The μCT scans were analyzed using the Dragonfly Software (Version 2022.2 for Windows. Object Research Systems (ORS) Inc, Montreal, Canada, 2020). Three-dimensional models of the scanned valves were created by the software using built-in segmentation tools (see Additional file 1: Figure S1 in the Supporting Information). The three-dimensional models were meshed in the software and exported as STL files. The median axes of the valves were found using the dense graph function and converted into STL file data. These files were then uploaded into MATLAB (The MathWorks Inc. (2022). MATLAB (version: 9.13.0 (R2022b), Natick, Massachusetts)) for further measurements and analysis (see Additional file 1: Figures S2, S3 in the Supporting Information). The processed scan STL files were converted into point clouds using the computer vision toolbox. A script was developed for fitting the point clouds to the geometric models. The mathematical model used for the script is further elaborated in the supporting information.

### Finite elements analysis

We used Abaqus-CAE (v.6.14, ABAQUS Inc., Ca, USA) for our finite element analysis.

The scanned dorsal and ventral valves used for the analyses were from females with no ovipositional history. The valves were meshed using C3D4 elements with at least 10^6^ elements for each model. We employed an isotropic and linear-elastic material model and analyze only geometrical differences while fixing the remaining parameters of the system. The elastic modulus was defined to be 5 GPa, based on measurements we conducted in our previous study [14], and the Poisson’s ratio was 0.38 [22]. The valves were positioned geometrically such that their tips coincided in angle as much as possible. The bases of the valves were fixed to restrict all degrees of freedom. Two types of simulations were performed on each valve, using two different forces applied at the tip. F_1_ (propagation) was applied in the direction of propagation and F_2_ (digging) in the direction of burrowing. For both valves, the same load of 5N was defined for all simulations and the same direction vectors were defined. This value corresponds to the maximal force that was applied by the female locust during digging, measured in our previous study [14]. We present the von Mises stress distributions, which indicate an effective quantity of the stress tensor that combines all the stress components (i.e., axial and shear in all directions). The von Mises stress distribution represents the typical stress magnitude at given points in the material, which may lead to mechanical failure when exceeding certain thresholds. In the shape variation simulations, two 3D geometrical models were created by fitting each design here to a dorsal valve of a locust with no oviposition history. The geometrical models were first created as 3D splines in MATLAB and then transferred to SolidWorks where the splines were used as an outline to create 3D shapes. The shapes were then transferred to Abaqus and meshed using C3D8R elements. The material was defined with the same properties as those used for the valve scans. The bases were fixed to restrict all degrees of freedom and three types of loads were applied to the geometrical models. In all cases, the total load applied was 5 N [14]. F_1_ and F_2_ are the forces applied to the tip of the models that correspond to the same forces applied to the valve scans. F_3_ is the pressure applied to the soil-shoveling surface of the valve in order to simulate the load that shoveled soil applies to the valve during digging.

### Supplementary Information

Additional file 1: Supporting Information. Figures S1–S3: geometrical modeling. Figures S4: Simulated mechanical responses. Figure S5. The digging valves in their open and closed states

## Data Availability

All study data are included in the article and/or supplementary information.
