# Supplementary material for: Asymmetry between the dorsal and ventral digging valves of the female locust: function and mechanics
Source: BMC Biol. 2024 May 31;22:129. doi: 10.1186/s12915-024-01930-0 (PMC11143638; doi:10.1186/s12915-024-01930-0)
Supplement: Supplementary file 1 — Additional file 1: Supporting Information. Figures S1–S3: geometrical modeling. Figures S4: Simulated mechanical responses. Figure S5. The digging valves in their open and closed states [file 12915_2024_1930_MOESM1_ESM.docx]

**Supporting Information**

**Asymmetry between the dorsal and ventral digging valves of the female locust: function and mechanics**

Shmuel Gershon^a^, Benny Bar-On^b^, Shai Sonnenreich,^a^ Amir Ayali^c^, Bat-El Pinchasik^a,d^

^a^ School of Mechanical Engineering, Tel-Aviv University, Tel-Aviv, 6997801, Israel

^b^ Department of Mechanical Engineering, Ben-Gurion University of the Negev, Beer-Sheva, 84105, Israel

^c^ School of Zoology, Faculty of Life Sciences and Sagol School of Neuroscience, Tel-Aviv University, Tel-Aviv, 6997801, Israel

^d^ Center for Physics and Chemistry of Living Systems, Tel Aviv University, Tel Aviv 69978, Israel

**Keywords**

Biomechanics, digging, finite element method, structure-function relationships, geometry

**Corresponding author**

pinchasik@tauex.tau.ac.il

*Geometrical Modelling*

The valves’ geometrical model was created with MATLAB R2022b. The processed scans STL files were turned into point clouds by using the partial differential equation toolbox and computer vision toolboxes in MATLAB. The geometric model consists of two main components: a 3D spline and a model describing the hemi-elliptic sections that are attached to a Frenet frame along the spline. The sections are attached to the spline at the center of the major axis of the hemi-ellipse, similarly to previous studies^1-7^.

In our model, we place the 3D spline at the center of the surface that comes in contact with the soil. Due to the complex three-dimensional shape of the valve surface, we used the projection of the medial axis on the surface of the 3D shape as an approximation of its centerline. Various algorithms for finding the medial axis of complex 3D shapes have been developed^8–12^. We used the built-in algorithm in the Dragonfly software^13^ to create medial axes for the valves as shown in **Figure S1**a.


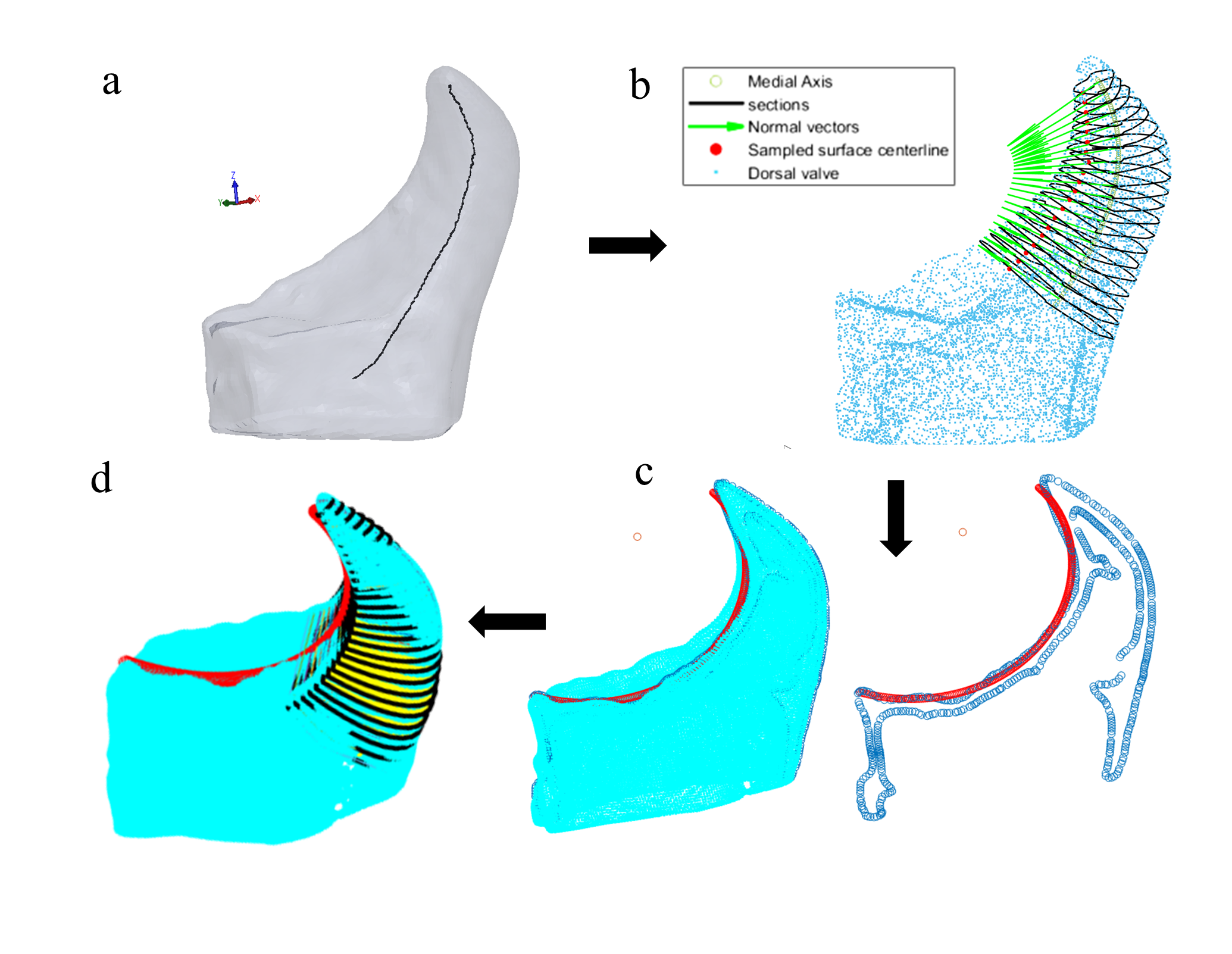


**Figure S1.** a) µCT scan of a dorsal valve with its medial axis. b) Sampling the surface centerline using the normal vector of the local Frenet frame of the medial axis. c) Section of the valve along a plane that runs through the sampled surface centerline. d) Model sections are placed on the local Frenet frame along the fitted centerline from base to tip.

We find the center of the surface on each section by finding the closest vertex to the normal vector of the medial axis as shown in Figure S1b. Combining the vertices from all of the sections generates a sampled line of points on the outside surface of the valve. In the case of the dorsal valve we discovered that these points can be fitted with a 2D surface which, in turn, can be used to take a slice out of the valve as shown in Figure S1c. The sampled points can be fitted using different curve functions (e.g. a logarithmic spiral) but for simplicity, a 3D second order polynomial spline was used (equations 1, **Table S1**):

1. $\vec{H}\left( \theta\right)= \left\{ \begin{aligned} x=(p1*t^{2}+p2*t+p3)\hat{x} \\ y=(p4*t^{2}+p5*t+p6)\hat{y} \\ z=(p7*t^{2}+p8*t+p9) \hat{z} \end{aligned} \right.$

*t* is an independent parameter ranging between 1 and 10. The point clouds of the valves were sectioned at constant intervals, perpendicular to the spline as shown in Figure S1d. The outer points of each section were used to create 2D section perimeters. The area of each respective perimeter was measured. The local radius of each section was calculated by equation (2) and together with *d(t)*- the length of $\vec{L}$ from *t_min_* up to *t*, yielded the growth rate of the sections along the spline.

1. $r(t)=\sqrt{\frac{SectionArea\left( t \right)}{\pi}}$
2. $r(t)=C_{Slope}d\left( t \right)+C_{Intercept}$

The sections were of roughly hemi-elliptical shapes and thus, the outer-shell, created along the centerline, was modeled using a hemi-ellipse. The hemi-ellipse was positioned such that the center of the full ellipse would be located on the centerline. The outline of the hemi-elliptical sections was measured in order to find the aspect ratio $AR$ between the major and minor axis of the spiral. It was found that the aspect ratio is approximately 2 for all sections.

The hemi-elliptical sections were skewed in respect to the symmetrical shape, and a skewing term was added as shown in equations (4)-(5). By plotting the axis of the ellipse at each section, the direction and skewing of the sections could be measured. The skewing was found to be roughly linear along the valve for the dorsal valve and roughly constant for the ventral valves.

1. $x^{'}=\cos\left( \omega\right)r(t)\vec{e}_{2}$
2. $y^{'}=\sin\left( \omega\right)r(t)AR\vec{e}_{3}$

*ω* is defined between the range: $0^{\circ}+\eta_{start}+\eta d\left( t \right)\leq\omega\leq180^{\circ}+\eta_{start}+\eta d\left( t \right)$.

Equations (6)-(8) show the alignment of the sections along the spline. The orientation of the axis of the hemi-ellipse at each section was defined by orienting the semi minor axis in the direction of the vector, normal to the gradient ($\vec{e}_{2}$).

1. $\vec{e}_{1}=\frac{\vec{L}_{t}(t)}{\left| \vec{L_{t}}(t) \right|}$
2. $\vec{e}_{2}=\frac{{\vec{e}_{1}}_{t}(t)}{\left| {\vec{e}_{1}}_{t}(t) \right|}$
3. $\vec{e}_{3}=\vec{e}_{1}\times\vec{e}_{2}$

The skew term was adjusted until the highest overall accuracy was achieved between the sections of the samples and the sections of the model (**Figure S2**).


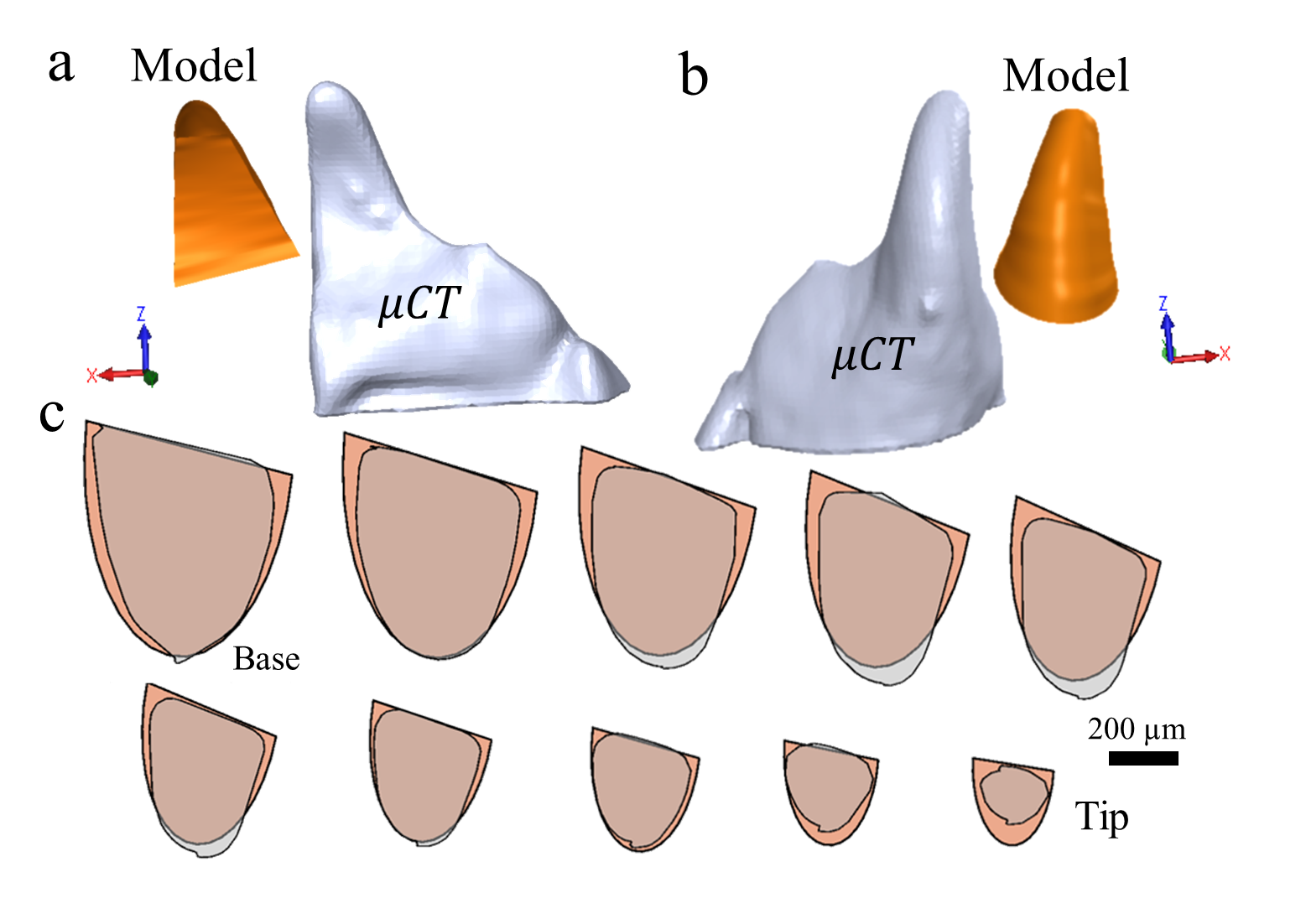


**Figure S2.** a) The analytical model (orange) and a 3D µCT model (grey) of a ventral valve are shown side by side from the a) outer and b) medial perspectives. c) Section overlays between the models.

Figures S2a,b show the µCT scan of the valve, and the corresponding model, from the dorsal and ventral views, respectively. The accuracy was measured for each section in the following way. First, we calculated the intersected area between the sample and model (Figure S2c). This area was divided once by the sample area and once by the model area. The average of those two results was defined as the section accuracy. The overall accuracy of the fit was calculated as the average of all the section accuracies.

**Figure S3** shows overlays between cross-sections of the model and the biological valves throughout the organ.


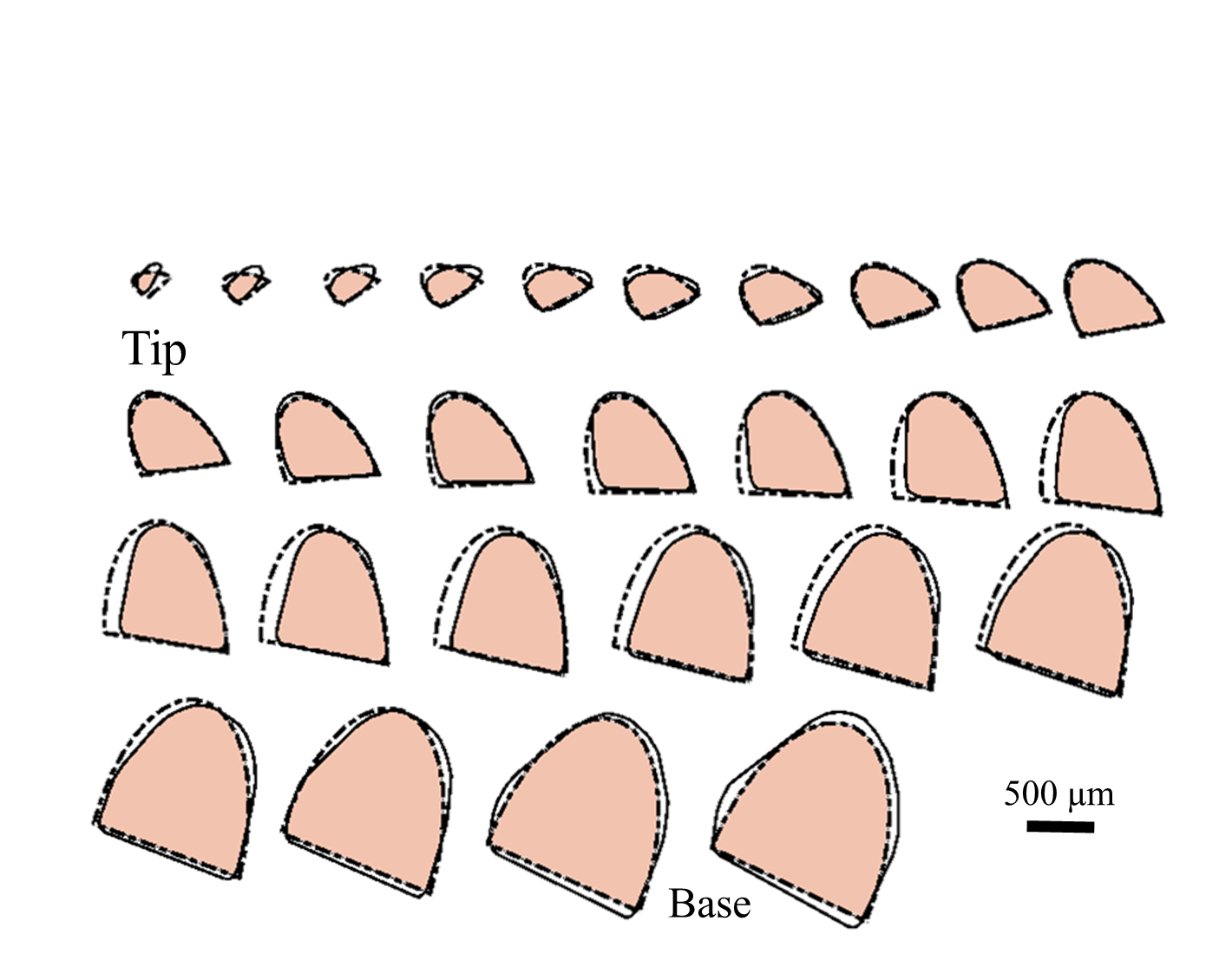


**Figure S3.** An overlay of $\mu$CT scan sections and model sections of an entire dorsal valve tip, taken from tip to base, at 0.064 mm intervals. Solid lines correspond to $\mu$CT slices. Dotted Lines correspond to fitted hemi-elliptical section at each slice. Colored area denotes the overlap between model fit and section. Total overlap between the model and scan is 92.8%.

| Centerline spline parameters | $p_{1}-p_{9}$ | Polynomial 3D spine parameters |
| --- | --- | --- |
|  | $t$ | Polynomial spline running parameter. |
| Section size growth parameters | $AR$ | The aspect ratio of the sections |
|  | $C_{slope}$ | the section size growth with distance slope along the spiral |
|  | $C_{Intercept}$ | section size function fit intercept |
| Section spin and skew | $\xi$ | section direction correction angle |
|  | $\eta$ | section skew linear change slope. |
|  | $\eta_{start}$ | Base section skew angle. |

**Table S1**. Model parameters.


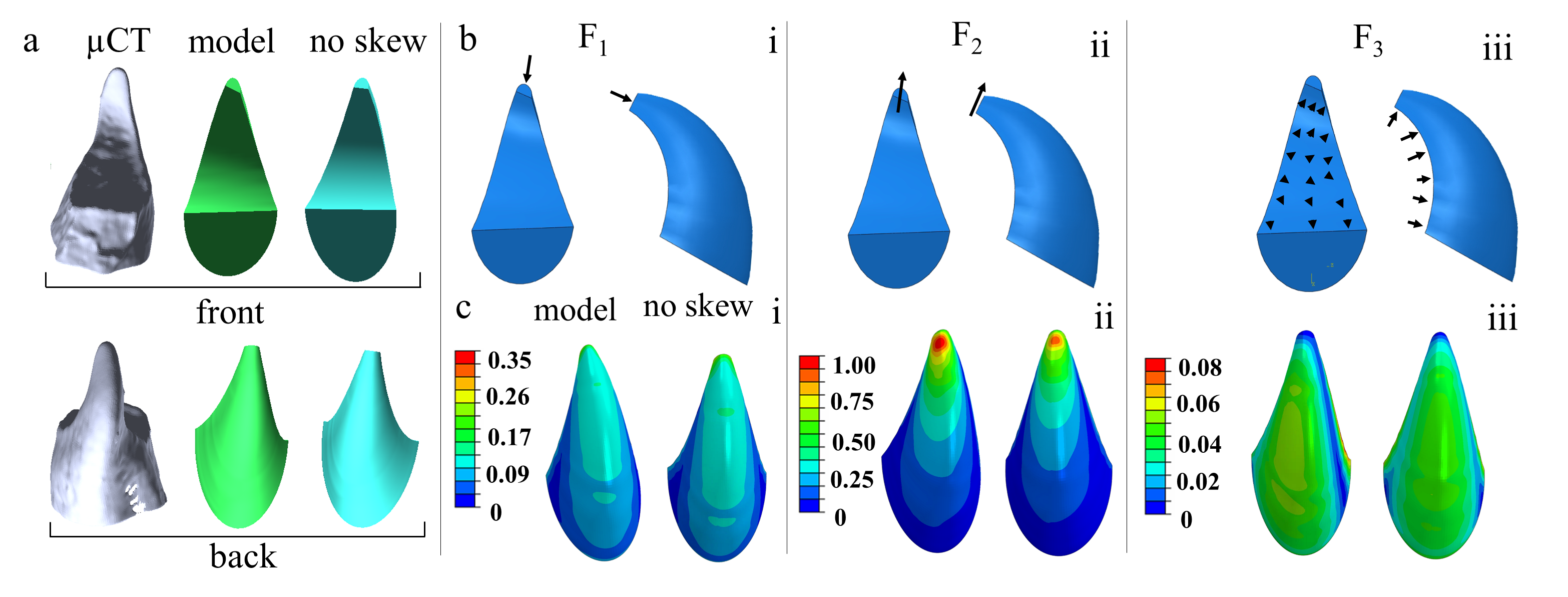
**Figure S4.** Mechanical response of valve models with varying shapes, at the proximal surface, which does not come in contact with the soil during digging. a) Computerized tomography scan of the dorsal valve (left), a model of the valve (middle), and the model without the skew (right). b) Applied forces in the direction of i) digging (F1), ii) propagation (F2), and iii) uniform distributed force (black arrows). c) Simulations showing the Von-Mises stress distribution in the valve model (left) and the model without the skew (right). For each load case a normalized stress scale bar is presented.


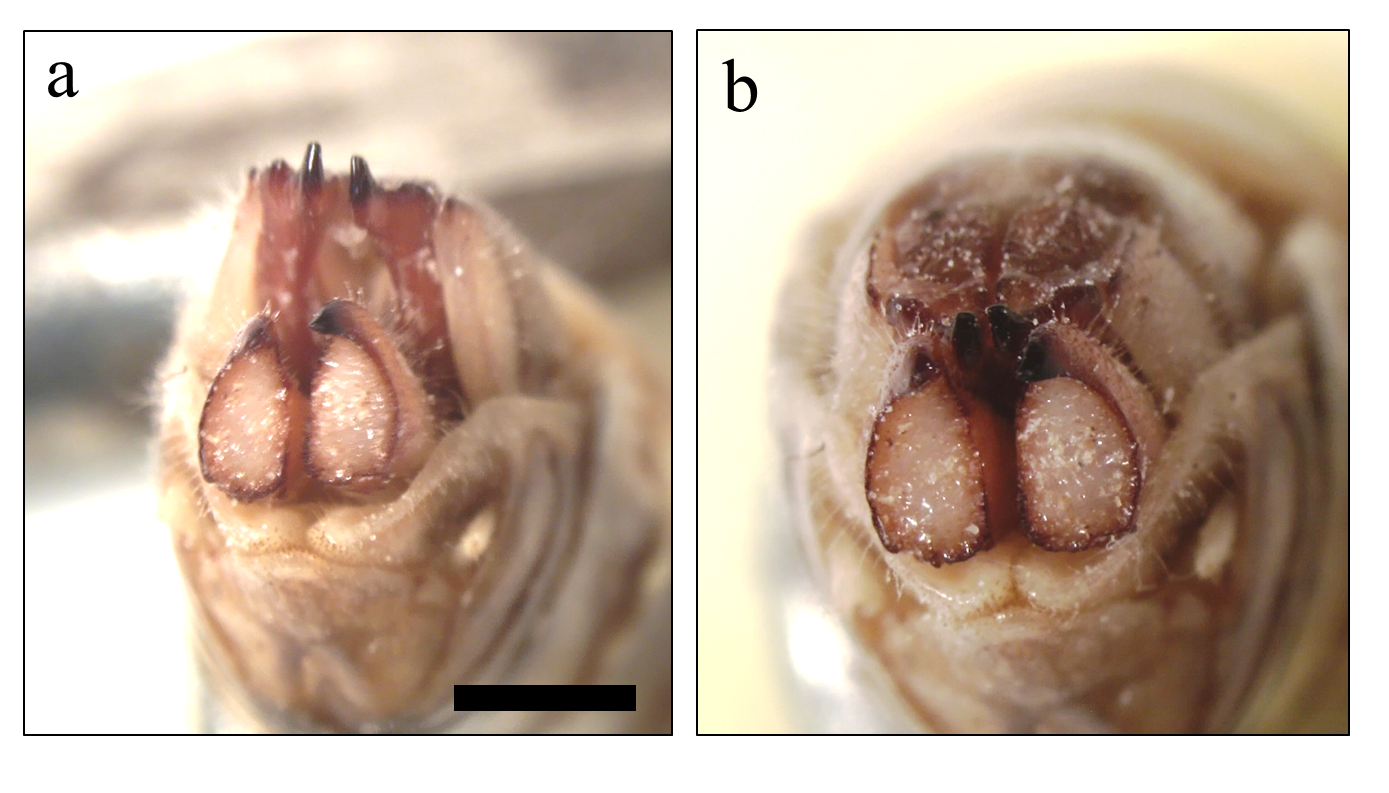


**Figure S5.** The digging valves in their a) open, and b) closed states. Scale bar corresponds to 4 mm.

**References**

(1) David M. Raup. Geometric Analysis of Shell Coiling: Coiling in Ammonoids. *Journal of Paleontology* **1967**, *41* (1), 43–65.

(2) Raup, D. M.; Michelson, A. Theoretical Morphology of the Coiled Shell. *Science (American Association for the Advancement of Science)* **1965**, *147* (3663), 1294–1295.

(3) Illert, C. Formulation and Solution of the Classical Seashell Problem. 21.

(4) Cortie, M. B. Digital Seashells. *Computers & Graphics* **1993**, *17* (1), 79–84. https://doi.org/10.1016/0097-8493(93)90054-D.

(5) Harary, G.; Tal, A. The Natural 3D Spiral. *Computer Graphics Forum* **2011**, *30* (2), 237–246. https://doi.org/10.1111/j.1467-8659.2011.01855.x.

(6) Evans, A. R.; Pollock, T. I.; Cleuren, S. G. C.; Parker, W. M. G.; Richards, H. L.; Garland, K. L. S.; Fitzgerald, E. M. G.; Wilson, T. E.; Hocking, D. P.; Adams, J. W. A Universal Power Law for Modelling the Growth and Form of Teeth, Claws, Horns, Thorns, Beaks, and Shells. *BMC Biol* **2021**, *19* (1), 58. https://doi.org/10.1186/s12915-021-00990-w.

(7) Faghih Shojaei, M.; Mohammadi, V.; Rajabi, H.; Darvizeh, A. Experimental Analysis and Numerical Modeling of Mollusk Shells as a Three Dimensional Integrated Volume. *Journal of the Mechanical Behavior of Biomedical Materials* **2012**, *16*, 38–54. https://doi.org/10.1016/j.jmbbm.2012.08.006.

(8) Au, O. K.-C.; Tai, C.-L.; Chu, H.-K.; Cohen-Or, D.; Lee, T.-Y. Skeleton Extraction by Mesh Contraction. *ACM Trans. Graph.* **2008**, *27* (3), 1–10. https://doi.org/10.1145/1360612.1360643.

(9) Cornea, N. D.; Silver, D.; Min, P. Curve-Skeleton Properties, Applications, and Algorithms. *IEEE Trans. Visual. Comput. Graphics* **2007**, *13* (3), 530–548. https://doi.org/10.1109/TVCG.2007.1002.

(10) Lin, C.; Li, C.; Liu, Y.; Chen, N.; Choi, Y.-K.; Wang, W. Point2Skeleton: Learning Skeletal Representations from Point Clouds. ***2021*** *IEEE/CVF Conference on Computer Vision and Pattern Recognition (CVPR)*; IEEE: Nashville, TN, USA, 2021; 4275–4284. https://doi.org/10.1109/CVPR46437.2021.00426.

(11) Tagliasacchi, A.; Zhang, H.; Cohen-Or, D. Curve Skeleton Extraction from Incomplete Point Cloud, *ACM Transactions on Graphics* **2009** 28,03,1-9

(12) Jayadevan, V.; Delp, E.; Pizlo, Z. Skeleton Extraction from 3D Point Clouds by Decomposing the Object into Parts. arXiv 26, **2019**. http://arxiv.org/abs/1912.11932.

(13) Dragonfly Software, Version 2022.2 for Windows. Object Research Systems (ORS) Inc, Montreal, Canada, 2020; Software Available at Http://Www.Theobjects.Com/Dragonfly.
